# Review of biomarkers in systemic juvenile idiopathic arthritis: helpful tools or just playing tricks?

**DOI:** 10.1186/s13075-016-1069-z

**Published:** 2016-07-13

**Authors:** Faekah Gohar, Christoph Kessel, Miha Lavric, Dirk Holzinger, Dirk Foell

**Affiliations:** Department of Paediatric Rheumatology and Immunology, University of Münster, Domagkstraße 3, D-48149 Münster, Germany

## Abstract

**Background:**

Diagnosing systemic juvenile idiopathic arthritis (SJIA) can be extremely challenging if typical arthritis is lacking. A variety of biomarkers have been described for the diagnosis and management of SJIA. However, very few markers have been well-validated. In addition, increasing numbers of biomarkers are identified by high throughput or multi-marker panels.

**Method:**

We identified diagnostic or prognostic biomarkers by systematic literature review, evaluating each according to a predefined level of verification, validation or clinical utility. Diagnostic biomarkers were those identifying SJIA versus (1) non-SJIA conditions or healthy controls (HC) or (2) other non-systemic JIA subtypes. Prognostic biomarkers were those specifically tested for the prediction of (1) disease flare, (2) increased disease activity +/- discrimination of active versus inactive disease, or (3) macrophage activation syndrome (MAS).

**Results:**

Fifty-five studies fulfilled the inclusion criteria identifying 68 unique biomarkers, of which 50/68 (74 %) were investigated by only a single research group. Candidate marker verification and clinical utility was evaluated according to whether markers were readily and reliably measurable, investigated by independent study groups, discovered by more than one method (i.e. verified markers) and validated in independent cohorts. This evaluation revealed diagnostic biomarkers of high interest for further evaluation in the diagnostic approach to SJIA that included heme oxygenase-1, interleukin-6 (IL-6), IL-12, IL-18, osteoprotegerin, S100 calcium-binding protein A12 (S100A12) and S100A8/A9.

**Conclusion:**

In summary, a number of biomarkers were identified, though most had limited evidence for their use. However, our findings combined with the identified studies could inform validation studies, whether in single or multi-marker assays, which are urgently needed.

**Electronic supplementary material:**

The online version of this article (doi:10.1186/s13075-016-1069-z) contains supplementary material, which is available to authorized users.

## Background

Systemic juvenile idiopathic arthritis (SJIA), or Still’s disease/syndrome, is a childhood rheumatic condition that is typically characterized by spiking fever in a quotidian pattern, transient rash and arthritis. Patients may alternate between periods of disease activity (flare) and inactive disease. SJIA accounts for around 10–20 % of juvenile idiopathic arthritis (JIA), which has an incidence of around 6.6–15 per 100,000 children [1]. Although defined as a subtype of JIA, patients often present with rather unspecific signs and symptoms initially, with the hallmark fever of unknown origin, but without chronic arthritis. Diagnosing SJIA is challenging in these cases as the disease is recognized as an autoinflammatory syndrome rather than classical autoimmune arthritis [2, 3]. Accordingly, most clinical symptoms can be attributed to dysregulated innate immune mechanisms with only minor involvement of adaptive immunity. Gene expression studies of circulating cells show increased levels of transcripts, reflecting monocyte/macrophage-associated activation in SJIA [4–6]. The innate immune cells such as monocytes and macrophages are thought to be drivers of SJIA, producing several mediators implicated in the pathogenesis of SJIA, including interleukin-1 (IL-1), IL-6 and IL-18 and phagocyte-specific S100 proteins [7]. IL-1 in particular seems to have a prominent role in SJIA. Serum from patients with SJIA induces the transcription of genes of the innate immune system including IL-1 in peripheral blood mononuclear cells (PBMC). Furthermore, activated monocytes from patients with SJIA secrete significantly more IL-1β in comparison with monocytes from healthy controls [6].

Significant challenges to improving the clinical care of patients with SJIA include the discrimination of SJIA from other causes of fever, evidence-based evaluation of response to treatment, detection and limitation of subclinical inflammation and discrimination of SJIA without macrophage activation syndrome (MAS) from SJIA with MAS [8]. MAS is a serious complication of SJIA with a 10 % mortality risk, defined as an acute episode of overwhelming inflammation and characterized by activation and expansion of T lymphocytes and hemophagocytic macrophages. In the early stages, development of MAS is difficult to predict and diagnostic and prognostic biomarkers might enable early intervention.

**Fig. 1:**
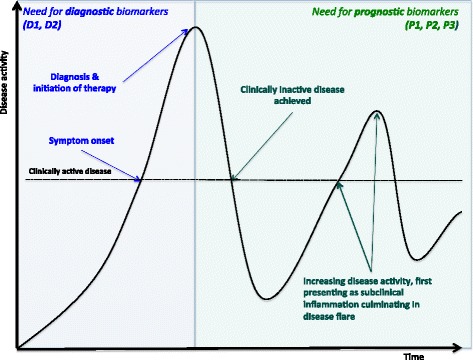
Biomarker need in clinical context. Typical clinical sequence in systemic juvenile idiopathic arthritis (SJIA) from disease onset, diagnosis to clinical resolution and flare. Specific time points where there is a need for diagnostic and prognostic biomarkers are indicated. Diagnostic markers are indicated as follows: *D1* SJIA versus other non-JIA conditions, *D2* SJIA versus other JIA subtypes. Prognostic markers are indicated as follows: *P1* prognostic for flare, *P2* prognostic for increased disease activity, *P3* prognostic for macrophage activation syndrome (MAS) or differentiating MAS from SJIA flare. Adapted from Hinze et al. 2015 [8]

These challenges could be addressed by the identification and validation of clinically relevant biomarkers, of which those circulating in serum and plasma are useful and easily obtainable from peripheral blood [9–13]. Mechanistic markers are those that are elevated or decreased in response to underlying pathological processes, whereas proxy markers, such as C-reactive protein (CRP), do not have a definite role in the pathology of the disease, and are non-specific markers of inflammation [14]. Therefore, measurement of a mechanistic biomarker can quantify a pathologic process. With such quantification, a level of severity can be defined, and cutoffs determined, allowing the use of such biomarkers as treatment targets (Fig. 1) [8, 15]. Diagnostic biomarkers, proxy or mechanistic, can aid detection of a disease or confirm it in uncertain cases e.g., evolving SJIA versus sepsis [15, 16].

Although a number of publications describe potential biomarkers, none have been recently validated or used in clinical studies aside from the IL-1 family cytokines and the S100-proteins, S100A12 and S100A8/A9 [17]. To date, discovery studies vastly outnumber validation studies, which are more challenging to perform given their requirement for independent cohorts and statistically valid sample sizes. Additionally, the number of identified candidates is usually large and the cost of validation high, leading to a need for unbiased prioritization of candidates for validation [18].

In conclusion, a combination of sensitive biomarkers could allow targeted and personalized treatment and improve treatment outcomes [8]. We therefore identified current candidate diagnostic and prognostic biomarkers from the literature, additionally evaluating their potential for validation/clinical use, function and association with other identified biomarkers. We also discuss the current and future potential of biomarkers for SJIA.

## Method

### Search criteria

A PubMed search was performed using the search terms as follows: "Arthritis, Juvenile“[Mesh] AND ((”2000/11/01"[PDAT]: “2015/11/01”[PDAT]) AND “humans”[MeSH Terms] AND English [lang]) along with the additional keywords: 1) cytokine (“cytokines”[MeSH Terms] OR “cytokines”[All Fields] OR “cytokine”[All Fields]) (*n* = 544 individual studies identified), OR 2) biomarker (“biological markers”[MeSH Terms] OR (“biological”[All Fields] AND “markers”[All Fields]) OR “biological markers”[All Fields] OR “biomarker”[All Fields]) (*n* = 307), OR 3) validation (*n* = 114). Abstracts of identified studies were reviewed and any fulfilling exclusion criteria at the outset were excluded, and the full text scrutinized for those remaining.

### Inclusion and exclusion criteria

Inclusion criteria were as follows: studies in which serum or plasma markers were analysed; original research studies; studies that specifically addressed the biomarkers with the diagnostic or prognostic functions as indicated in Table 1 and studies that also included SJIA-specific analyses. Exclusion criteria were: case studies or review articles; studies that included fewer than three patients with SJIA; studies with only negative findings reported (i.e. no statistically significant finding for the candidate marker for the use evaluated) and studies describing functional/cell-based assays or enzyme activity assays. We also excluded studies on adult-onset Still’s disease (AOSD) [19] and genetic array/genotype or phenotype studies that described individual patients rather than disease signatures, without evaluation of individual biomarkers, even if performed as unbiased discovery studies. Genetic markers and gene expression profiles in SJIA have been previously discussed in a review by Nirmala et al. [20].

**Table 1:** Diagnostic and prognostic criteria for inclusion

Biomarker function	Description	Biomarkers identified (*n*)	Number of studies in which biomarkers were identified (*n*)
Diagnostic	D1: SJIA versus other non-arthritis conditions or HC	36	48
	D2: SJIA versus other JIA subtypes	25	25
Prognostic	P1: for flare (or relapse)	14	16
	P2: for increased disease activity and/or the discrimination of active and inactive disease	15	21
	P3: for MAS or discriminating MAS from SJIA flare	7	12

### Data analysis and categorisation of biomarkers

Details recorded from identified studies included the aims, numbers of included patients and methods of biomarker assessment (Additional file 1). Biomarkers from each study were categorised as diagnostic (discriminating SJIA from non-JIA disorders or healthy controls (HC) termed “D1 biomarkers” or differentiating SJIA from other JIA subtypes, “D2 biomarkers”) or prognostic (for flare, “P1 biomarkers”, increased disease activity or discriminating active versus inactive disease, “P2 biomarkers”, or prognostic for MAS or differentiating SJIA with and without MAS, “P3 biomarkers”), as defined in Table 1, according to the study objectives, and indicated in Fig. 1.

### Evaluation of identified markers

Identified candidate biomarkers were scored and ranked by their potential to reach validation or clinical use, with potentially spurious or unreproducible candidate findings ranked the lowest. The biomarker scoring system (BMS) used (Table 2) was developed to identify whether identified candidates (1) were readily measurable, i.e. in standard collected biological samples and without special equipment, (2) had been measured by independent study groups, as confirmation that the biomarker is detectable, (3) had been discovered by more than one method, e.g., proteomic and enzyme-linked immunosorbent assay (ELISA) methods, (4) had been measured by an established assay, i.e. an assay that is well-described, with normal cutoff values available, as this would allow easier translation to clinical practice and finally (5) had been validated for the stated clinical question. Each evaluation question making up the BMS (Table 2) was answered using only the information collected during the review process, and each of the included five questions was scored 0 or 1.

**Table 2:** Scoring system used to perform an unbiased evaluation of identified biomarkers

Q1	Readily measurable (e.g. in serum)	Yes = 1	No = 0
Q2	Measured by more than one independent study group	Yes = 1	No = 0
Q3	Discovered by more than one single method	Yes = 1	No = 0
Q4	Measured by a reproducible assay	Yes = 1	No = 0
Q5	Validated in a validation cohort	Yes = 1	No = 0
	Maximum score = 5, minimum score = 0		

## Results and discussion

### Identified candidate biomarkers

A total of 57 studies describing 68 unique biomarkers were identified (Table 3). All reported biomarkers were identified in serum unless otherwise indicated (Additional file 1: Table S1). The mean number of patients with SJIA included in studies was 21 (range 4–60). There were 50 biomarkers (74 %) investigated in studies performed by a single research group and 29/57 studies evaluated a single biomarker. Biomarkers included cytokines, soluble receptors, antibodies, alarmins and other functional molecules (Table 3). The most studied biomarkers were IL-18 (*n* = 7 individual studies), IL-6 (*n* = 5), S100A8/A9 (*n* = 5), S100A12 (*n* = 4) and soluble CD25 (IL-2 receptor) (*n* = 4) (Table 3). Only two identified biomarkers, namely S100A8/A9 and S100A12, were described in JIA (but not SJIA) validation studies [21]. Hepcidin, also included as a diagnostic marker, was validated for differentiating SJIA-associated anaemia from anaemia of other causes, but not specifically for SJIA diagnosis [22].

**Table 3:** Identified serum and plasma biomarkers

Biomarker		Detection method	Intended use (P/D) + Reference		BMS score^a^ Q1 + Q2 + Q3 + Q4 + Q5 = total
Abbreviation/gene name	Full/alternative name		D	P	
A2M	Alpha-2-macroglobulin	Commercial ELISA		[45]	1 + 0 + 0 + 1 + 0 = 2
AB-oxLDL	Antibodies to oxidized low-density lipoprotein	Commercial ELISA	[68]		1 + 0 + 0 + 1 + 0 = 2
ACAN	Aggrecan core protein, cartilage-specific core protein	Immunoassay	[69]		1 + 0 + 0 + 1 + 0 = 2
ACPA	Anti-citrullinated protein antibodies	Commercial ELISA	[39]	[39]	1 + 0 + 0 + 1 + 0 = 2
ACT	Alpha-1-antichymotrypsin	Commercial ELISA		[45]	1 + 0 + 0 + 1 + 0 = 2
AECA	Anti-endothelial cell antibodies	In-house ELISA	[70]		1 + 0 + 0 + 1 + 0 = 2
AGP1	Alpha-1-acid-glycoprotein	Commercial ELISA		[45]	1 + 0 + 0 + 1 + 0 = 2
ANA	Antinuclear antibody	Fluorescence assay		[36]	1 + 1 + 0 + 1 + 0 = 3
		Commercial ELISA		[37]	
Anti-BiP	Anti-immunoglobulin binding protein/glucose regulated protein 78 (GRP78)	In-house ELISA	[71]		1 + 0 + 0 + 1 + 0 = 2
Anti-CCP	Anti-cyclic citrullinated peptide	Commercial ELISA	[72]		1 + 0 + 0 + 1 + 0 = 2
APO A1	Apolioprotein A1	Commercial ELISA		[45]	1 + 0 + 0 + 1 + 0 = 2
APO VI	Apolipoprotein VI	Commercial ELISA		[45]	1 + 0 + 0 + 1 + 0 = 2
APRIL	A proliferation-inducing ligand	Commercial ELISA	[73]	[73]	1 + 0 + 0 + 1 + 0 = 2
B2M	Beta -2-microglobulin	Not indicated		[74]	1 + 0 + 0 + 1 + 0 = 2
BAFF	B-cell activating factor	Commercial ELISA	[73]	[73]	1 + 0 + 0 + 1 + 0 = 2
C4	Complement C4	Commercial ELISA		[45]	1 + 0 + 0 + 1 + 0 = 2
CCL3	Chemokine (C-C motif) ligand 3	Luminex assay	[57]		1 + 0 + 0 + 1 + 0 = 2
CD10	Cluster of differentiation antigen 10, also called neprilysin	Fluorimetric assay	[75]		1 + 0 + 0 + 1 + 0 = 2
CFH	Complement factor H	Commercial ELISA		[45]	1 + 0 + 0 + 1 + 0 = 2
COMP	Cartilage oligomeric matrix protein	Commercial ELISA	[39]	[39]	1 + 1 + 0 + 1 + 0 = 3
			[76]	[77]	
				[78]	
CXCL9	Chemokine (C-X-C Motif) ligand 9	Luminex assay	[57]		1 + 0 + 0 + 1 + 0 = 2
Fibrin D-dimer		Commercial assay		[79]	1 + 0 + 0 + 1 + 0 = 2
FSTL-1	Follistatin-like protein 1	Commercial ELISA	[80]	[80]	1 + 1 + 0 + 1 + 0 = 3
				[81]	
GHRL	Ghrelin, appetite regulating hormone	Commercial ELISA	[82]		1 + 0 + 0 + 1 + 0 = 2
GSN	Gelsolin	Commercial ELISA		[45]	1 + 0 + 0 + 1 + 0 = 2
Hepcidin	Peptide hormone, released by hepatocytes	Commercial assay	[22]		1 + 0 + 0 + 1 + 0 = 2
HMGB1	High mobility group box protein 1	Commercial assay	[83]		1 + 0 + 0 + 1 + 0 = 2
HO-1	Heme oxygenase-1	Commercial ELISA	[84]	[85]	1 + 1 + 0 + 1 + 1 = 4
HP	Haptoglobin	Commercial ELISA		[45]	1 + 0 + 0 + 1 + 0 = 2
IFNg	Interferon gamma	Commercial ELISA	[86]	[86]	1 + 0 + 0 + 1 + 0 = 2
IgA RF	Ig A rheumatoid factor isotype	In-house ELISA	[87]		1 + 0 + 0 + 1 + 0 = 2
IgM RF	Ig M rheumatoid factor isotype	In-house ELISA	[87]		1 + 0 + 0 + 1 + 0 = 2
IL-10	Interleukin-10	Commercial ELISA	[88]	[85]	1 + 0 + 0 + 1 + 0 = 2
IL-12	Interleukin-12	Luminex assay	[57]		1 + 1 + 1 + 1 + 0 = 4
		Commercial ELISA	[89]		
IL-18	Interleukin-18	Commercial assay Luminex assay	[25]		1 + 1 + 1 + 1 + 0 = 4
			[57]	[24]	
			[86]	[90]	
			[90]	[91]	
			[92]		
IL-18BP	Interleukin-18 binding protein	Commercial assay	[86]		1 + 1 + 0 + 1 + 0 = 3
			[90]	[90]	
IL-1b	Interleukin-1beta	Commercial ELISA	[89]		1 + 0 + 0 + 1 + 0 = 2
IL-6	Interleukin-6	Luminex assay Commercial ELISA	[57]	[24]	1 + 1 + 1 + 1 + 0 = 4
			[86]		
			[89]		
			[76]		
IP-10/CXCL10	IFNg-induced protein 10, or C-X-C motif chemokine 10	Commercial ELISA Luminex assay	[57]		1 + 1 + 0 + 1 + 0 = 3
			[86]		
			[93]		
LGALS3	Galectin-3	Commercial ELISA	[94]		1 + 0 + 0 + 1 + 0 = 2
MIF	Macrophage migration inhibitory factor	Luminex assay	[57]		1 + 0 + 0 + 1 + 0 = 2
MMP-3	Matrix metalloprotinease-3/stromelysin-1 (SL-1)	Commercial ELISA	[72]		1 + 0 + 0 + 1 + 0 = 2
Neopterin		Commercial ELISA		[85]	1 + 0 + 0 + 1 + 0 = 2
NO	Nitric oxide	Spectrophotometry		[95]	1 + 0 + 0 + 1 + 0 = 2
OPG	Osteoprotegerin/TNF 11B	Luminex assay Commercial ELISA	[57]		1 + 1 + 1 + 1 + 0 = 4
			[96]		
OPN	Osteopontin, phosphoglycoprotein	Commercial ELISA	[97]		1 + 0 + 0 + 1 + 0 = 2
RA33	Anti-heterogeneous nuclear ribonucleoprotein A2 antibodies	Commercial ELISA	[98]		1 + 0 + 0 + 1 + 0 = 2
RANKL	TNF ligand superfamily member 11/receptor activator of nuclear factor kappa B ligand	Commercial ELISA	[96]		1 + 0 + 0 + 1 + 0 = 2
Resistin	Protein adipokine	Commercial ELISA	[99]		1 + 0 + 0 + 1 + 0 = 2
S100A12	S100 calcium-binding protein A12	In-house ELISA Commercial ELISA	[45]	[45]	1 + 1 + 1 + 1 + 0 = 4
			[100]	[100]	
			[101]	[102]	
S100A8/A9	MRP8/14 (myeloid regulatory protein 8/14) complex, complex of S100A8 (Calgranulin A) and S100A9 (Calgranulin B)	In-house ELISA Commercial ELISA	[23]		1 + 1 + 1 + 1 + 0 = 4
			[45]	[23]	
			[101]	[17]	
			[103]	[45]	
SAA	Serum amyloid A	Commercial ELISA	[45]	[45]	1 + 1 + 0 + 1 + 0 = 3
			[76]		
SAP	Serum amyloid P	Commercial ELISA		[45]	1 + 0 + 0 + 1 + 0 = 2
sCD163	Soluble cluster of differentiation 163/haemoglobin scavenging receptor	Commercial ELISA		[85]	1 + 1 + 0 + 1 + 0 = 3
				[104]	
sCD21	Soluble cluster of differentiation 21	Commercial ELISA	[105]		1 + 0 + 0 + 1 + 0 = 2
sCD23	Soluble cluster of differentiation 23/soluble low affinity immunoglobulin epsilon Fc receptor)	Commercial ELISA	[105]		1 + 0 + 0 + 1 + 0 = 2
sCD25	Soluble cluster of differentiation 25/soluble interleukin-2 receptor alpha	Commercial ELISA		[74]	1 + 1 + 0 + 1 + 0 = 3
				[104]	
				[106]	
				[107]	
sE-selectin	Soluble E-selectin adhesion molecule	Commercial ELISA	[108]		1 + 1 + 0 + 1 + 0 = 3
			[109]		
			[110]		
sICAM-1	Soluble intracellular adhesion molecule-1	Commercial ELISA	[108]		1 + 1 + 0 + 1 + 0 = 3
			[109]		
			[110]		
sRAGE	Soluble receptor for advanced glycation end products	Commercial assay	[83]		1 + 0 + 0 + 1 + 0 = 2
sST2	Soluble ST2, also called interleukin 1 receptor-like 1 (IL-1RL1)	Commercial ELISA	[111]	[111]	1 + 0 + 0 + 1 + 0 = 2
sTM	Soluble thrombomodulin/CD141	Commercial ELISA	[112]		1 + 0 + 0 + 1 + 0 = 2
sTNFR55	Soluble tumour necrosis factor receptor 55	Commercial ELISA	[113]		1 + 0 + 0 + 1 + 0 = 2
sTNFR75	Soluble tumour necrosis factor receptor 75	Commercial ELISA	[113]		1 + 0 + 0 + 1 + 0 = 2
Survivin		Commercial ELISA	[76]		1 + 0 + 0 + 1 + 0 = 2
TIMP	Tissue inhibitors of metalloproteinases	Commercial ELISA	[96]		1 + 0 + 0 + 1 + 0 = 2
TNF-alpha	Tumour necrosis factor-alpha	Commercial ELISA	[88]		1 + 0 + 0 + 1 + 0 = 2
TTR	Transthyretin	Commercial ELISA	[45]	[45]	1 + 0 + 0 + 1 + 0 = 2

^a^Biomarker scoring system (BMS) biomarker score: each answer is scored as follows: yes = 1, no = 0. *D* diagnostic, *P* prognostic, *Q1* readily measurable (e.g. in serum), *Q2* measured by more than one independent study group, *Q3* discovered by more than one single method, *Q4* measured by a reproducible assay, *Q5* validated in a validation cohort, *IFN* interferon, *TNF* tumour necrosis factor

### Current clinical uses of identified biomarkers

This study identified some well-established markers of inflammation and/or SJIA, such as the S100-proteins (S100A12 and S100A8/A9 complex), IL-18 and IL-6, autoantibodies, non-specific inflammatory markers and some markers not classically associated with SJIA, such as B cell markers. S100A8/A9 is a predictive biomarker for subclinical disease activity and a predictor of JIA relapse after stopping medication [17, 21, 23]. IL-18 concentration is a known marker of disease activity in SJIA, while IL-18 and IL-6 can define subsets of SJIA [24–26]. While IL-6 and IL-1 are targets of the biological therapies tocilizumab, canakinumab, rilonacept and anakinra, respectively, neither cytokine is routinely measured in patients [27–30]. IL-1b, as already discussed, is usually undetectable in serum and IL-18 is not regularly measured due to technical limitations in performing bioassays [31]; however the reason for IL-6 not being used in routine care is unclear [32, 33].

A number of autoantibodies were identified as candidate biomarkers, including rheumatoid factor (RF), antinuclear antibodies (ANA) and anti-citrullinated protein antibodies (ACPA). RF has long been recognised as distinguishing RF-positive and RF-negative forms of polyarthritis (JIA subtypes) [34]. ANA are routinely evaluated in JIA as a screening factor for JIA-associated uveitis [35]. However, Shin et al. showed that ANA levels can change over time in patients with SJIA, which is a finding replicated by Huegle et al. [36, 37] ACPA are associated with joint damage, and are included in the classification criteria for rheumatoid arthritis, though they do not have an established use in SJIA [38–40].

CRP and ferritin, which are routinely measured, non-specific, acute phase reactants used as surrogate markers, were described as baseline parameters in most of the identified studies, but were not the subject of investigation in the studies, so were therefore excluded from Tables 1 and 3 and from further analyses. Other non-specific identified biomarkers of inflammation were serum amyloid (SAA), fibrin D-dimer and complement 4 (C4). While previously important in detecting long-term complications of inflammation such as amyloidosis, SAA measurement has become less important since the introduction of biological treatments, which have reduced complications in SJIA.

### Candidate biomarkers categorised as diagnostic or prognostic

Some biomarkers were identified in more than one study as described above and evaluated for more than one clinical question (Tables 1 and 3). There were 51 markers characterised as diagnostic, 33 as prognostic and 16 were both diagnostic and prognostic (Table 3): these were ACPA, A proliferation-inducing ligand (APRIL), B-cell activating factor (BAFF), cartilage oligomeric matrix protein (COMP), follistatin-like protein 1 (FSTL-1), heme oxygenase-1 (HO-1), interferon gamma (IFNg), IL-10, IL-18, IL-18 binding protein (IL-18BP), IL-6, S100A12, S100A8/A9, SAA, soluble ST2/IL-1 receptor-like 1 (sST2) and transthyretin (TTr).

### Evaluation of identified markers by clinical question

**Fig. 2:**
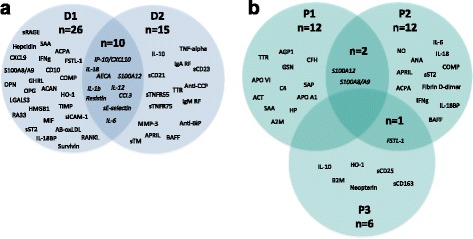
Identified biomarkers grouped by clinical question. **a** Diagnostic biomarkers are shown that differentiated systemic juvenile idiopathic arthritis (SJIA) from healthy controls (HC) or other non-JIA disease (*D1*), SJIA vs other JIA subtypes (*D2*) or both (*D1* and *D2*). **b** Prognostic biomarkers for flare (*P1*), increased disease activity or discriminating active disease from inactive (*P2*), for macrophage activation syndrome (MAS) or discriminating MAS from SJIA flare (*P3*), or a combination of these are shown. The specific clinical question is very important in interpreting the results of biomarker studies. Little overlap between different diagnostic questions suggests a predominance of different pathways during different stages of disease and therefore a specific hypothesis and clinical question is more useful in studies to understand mechanisms. Biomarkers that are broad enough to cover more than one diagnostic or prognostic category may be more likely to have a specific role in the underlying immunological pathology, and as broad markers will be more useful for wider clinical care. By performing this analysis we can create a shortlist of biomarkers on which to focus. Indeed, only a few markers fall into this group, but perhaps they should receive most attention for future validation in preference to other markers. *ACAN* aggrecan core protein cartilage-specific core protein, *ACCP* anti-cyclic citrullinated peptide, *ACPA* anti-citrullinated protein antibodies, *ACT* alpha-1-antichymotrypsin, *AECA* anti-endothelial cell antibodies, *ANA* antinuclear antibodies, *Anti-BiP* anti-immunoglobulin binding protein/glucose regulated protein 78 (GRP78), *APO* apolioprotein, *APRIL* A proliferation-inducing ligand, *B2M* Beta -2-microglobulin, *BAFF* B-cell activating factor, *COMP* cartilage oligomeric matrix protein, *CRP* C-reactive protein, *FSTL-1* follistatin-like protein 1, *GSN* Gelsolin, *HO-1* heme oxygenase-1, *IFN* interferon, *IL-18BP* IL-18 binding protein, *LGAL* galectin, *MMP* matrix metalloproteinase, *ONP* osteopontin, *SAA* serum amyloid A, *SAP* serum amyloid P, *sICAM-1* soluble intracellular adhesion molecule-1, *sST2* soluble ST2/IL-1 receptor-like 1, *TIMP* tissue inhibitors of metalloproteinase, *TTr* transthyretin

There were 36 biomarkers that differentiated SJIA from HC or other non-JIA disease (D1 biomarkers) and 25 markers differentiated SJIA from other JIA subtypes (D2 biomarkers). Of the prognostic markers, 14 P1 (flare), 15 P2 (disease activity) and seven P3 (MAS) biomarkers were identified (Table 1). Ten biomarkers were common to D1 and D2 (Fig. 2a); however, few markers overlapped between the prognostic groups (Fig. 2b). This analysis suggests that some biomarkers could have broad use as diagnostic or prognostics markers, rather than being useful only for specific questions. These markers might therefore be more useful than others in a clinical setting, and might therefore be prioritised for validation.

### Evaluation of candidate markers

For unbiased and valid results, biomarker evaluation should be performed according to a predefined hypothesis [41] in order to identify candidates more likely to be specific, rather than a high number of unspecific candidates. High throughput methods are increasingly sensitive and producing ever larger numbers of candidate biomarkers; however, they can still be impeded by methodological limitations, such as in LC-MS/MS, by the presence of high abundant proteins [42, 43]. Therefore, careful and evidence-based hypothesis-driven evaluation and prioritisation of candidates for validation studies is vital. While discovery studies are usually unbiased, the prioritisation of identified markers for further evaluation is much more variable, and might be reported as being based on reproducibility, availability of antibodies or levels of protein expression [44]. However, too often these data are omitted, leading to bias in the selection procedure. Ling et al. detected 26 proteins in plasma from patients with SJIA, which differentiated flare from quiescence plasma, of which 18 proteins were significant, and from these the top 15 were selected for unsupervised analysis and shown to remain significant [45]. However, only a limited panel of 9/15 were further tested, chosen according to the availability of antibodies and ELISA. As there is no quantitative and unbiased approach for prioritising candidate markers, we created the novel but unvalidated BMS (Table 2) for this study.

We evaluated each identified biomarker (Table 3) using the BMS (Table 2). No biomarker had the maximum score (5/5). The highest-scoring markers (score 4/5) were HO-1, IL-6, IL-12, IL-18, osteoprotogerin (OPG), S100A12 and S100A8/A9 (*n* = 7). There were 10 and 51 biomarkers with scores of 3/5 and 2/5, respectively. A score of 3/5 or greater, therefore, identified 17 (25 %) of the total biomarkers. The highest-scoring markers grouped according to diagnostic or prognostic subgroup are indicated in Fig. 1.

Next, the 36 identified D1 biomarkers, the largest group of identified biomarkers for any of the clinical questions asked, were scored and ranked as an example to show how the BMS could prioritise candidates for further evaluation (shown in Fig. 2, scores in Table 3). Seven biomarkers scored 4/5 (as listed above) and seven others scored 3/5, while the remaining 22 markers scored 2/5. This resulted in a panel of 14 markers when the cutoff was applied at a score of 3/5 or above (or *n* = 15 when S100A8 and S100A9 were analysed as separate proteins). Further ranking of markers within these broad groups was not performed. The online Search Tool for the Retrieval of Interacting Genes/Proteins (STRING) platform was used to identify if any of these 15 proteins had known functions in common [46]. To differing extents STRING identified direct or indirect functional link or interactions between all proteins except S100A12, FSTL-1 and COMP (when tested at the “medium” confidence level). All proteins were identified to be extracellular, consistent with their measurement in peripheral blood, and had an identified immune function role. A summary of the functions of this protein set is shown in Additional file 2: Table S2.

### The biomarker panel approach

Multiplex cytokine analysis can (1) differentiate SJIA from differential diagnoses and (2) identify distinct profiles in individual patients. Identification of cytokine patterns in individual patients could lead to the identification of subphenotypes within SJIA and also provide insight into the underlying biological basis for the clinical heterogeneity seen in SJIA [47, 48]. This clinical variation and the variety in identified biomarkers supports a prevailing view that a biomarker panel is required [8]. A “multimarker approach” is already used to predict risk of cardiovascular events and the multibiomarker assessment of disease activity (MBDA) is validated for rheumatoid arthritis (RA) [49–55]. The MBDA outperforms clinical assessment alone, imaging and single biomarker measurement, and is also cost-effective, measuring 12 biomarkers in just 0.2 ml of serum. A potential panel of biomarkers has recently been identified for paediatric systemic lupus erythematosus, which had good predictive value for detecting the complication lupus nephritis [56]. Jager et al. identified cytokine profiles in paired plasma and synovial fluid samples in 20 patients with SJIA using a bead array based multiplex immunoassay which measured 30 soluble inflammatory mediators in only 50 μl of sample and showed the blind measurement of IL-18 predicted patients with active SJIA with 93 % accuracy [57, 58]. While the identified studies in our analysis often evaluated more than one candidate marker, combinations of markers were not tested and did not feature in study hypotheses/design and/or sample numbers.

### Prerequisites for clinical biomarkers

Sample-specific and method-specific factors should be considered before performing either discovery or validation studies [59]. Sample requirements differ according to the planned methodology and platform to be used [60, 61]. Some cytokines, such as IL-1β, are extremely sensitive to degradation by freeze-thawing, whereas IL-18 is comparatively more stable [61]. A clinical biomarker should also fulfil an unmet need and improve existing tests, while also being cost-effective, criteria which will also help define candidates for validation [10, 59, 62]. We did not investigate the cost-effectiveness of markers. However, the validation and clinical use of many of the biomarkers, as described, is limited by the cost and/or local availability of diagnostic tests.

### Validation of biomarkers

Most candidate markers (86 %) were identified in a single study and/or by a single group, respectively. While this indicates that multiple groups are working on SJIA biomarkers, each with different strategies, it also reflects a lack of current understanding of the pathology of SJIA. Methods of biomarker verification, as intermediary steps towards validation, become increasingly important as new and improved biomarker discovery techniques result in large numbers of candidates [35, 63]. Identification of the same biomarker by multiple research groups could be seen as a verification step, suggesting a false positive finding to be less likely. Other verification factors might include confirmation that a candidate biomarker can be robustly measurable in peripheral blood, or the use of specific verification methods such as proteomic mass-spectrometry-based selected reaction monitoring (SRM) analysis [18]. SRM measures multiple target proteins, identified from discovery studies or existing literature simultaneously, without requiring specific antibodies as with antibody-based validation techniques, but it does not replace validation.

Biomarker validation, most frequently performed using antibody-based assays, is a difficult, costly and time-consuming process [35]. An example of validation is the included study by Rothmund et al. which compared different assays for measuring S100-proteins in JIA [21, 64]. Biomarker validation, also termed “qualification”, can be separated from clinical validation as a process referring more specifically to the process of linking biomarkers to a clinical endpoint based on evidence and statistical analysis [65]. Validation is widely acknowledged to be a more difficult process than identification, due to the requirement of large numbers of samples of well-defined patients from populations not used in the discovery step. An example use of a validated diagnostic biomarker or panel could allow earlier diagnosis of SJIA, allowing treatment to be started during the “window of opportunity”, the time point early enough in disease that intensive targeted treatment could be used to achieve early disease remission [8, 29, 66]. We therefore focused on identifying potentially clinically relevant diagnostic or prognostic biomarkers for SJIA from studies addressing specific clinical questions.

## Conclusions

There remains a need for the simultaneous evaluation of multiple biomarkers and an unbiased method of selecting candidate biomarkers for further evaluation. The parallel use of different methodological platforms such as microbead arrays (e.g. Luminex xMAP), aptamer-based assay or label-free liquid mass spectrometry (LC-MS/MS) could improve the spectrum of detected proteins [67], while the BMS used here is an example of how candidate markers could be prioritised. Markers that exclude SJIA would also be useful in the clinical setting. In particular, markers diagnostic for the main differential diagnoses of SJIA, such as the causes of fever of unknown origin, which might include infection or malignancy, would help exclude SJIA as a diagnosis. While this review was not designed to explore markers of differential diagnoses of SJIA, including them in a potential multi-marker panel would likely improve such a diagnostic assay.

Sixty-eight unique candidate markers evaluated for the management of SJIA were identified by this literature review. Only one identified study was a validation study and very few identified biomarkers were evaluated by more than one study group. Therefore, there is a clear and urgent need to confirm and consolidate findings from discovery studies and validate findings. The use of emerging technologies, with collaborative efforts, may ultimately help achieve the goal of validating new diagnostic or prognostic biomarkers, or panels of biomarkers, for improving the management of SJIA.

## Abbreviations

ACPA, anti-citrullinated protein antibodies; ANA, antinuclear antibodies; APRIL, A proliferation-inducing ligand; BAFF, B-cell activating factor; BMS, biomarker scoring system; COMP, cartilage oligomeric matrix protein; CRP, C-reactive protein; ELISA, enzyme-linked immunosorbent assay; FMF, familial Mediterranean fever; FSTL-1, follistatin-like protein 1; HC, healthy controls; HO-1, heme oxygenase-1; IFN, interferon; IL, interleukin; IL-18BP, IL-18 binding protein; JIA, juvenile idiopathic arthritis (non-systemic); LC-MS/MS, label-free liquid mass spectrometry; MAS, macrophage activation syndrome; MBDA, multibiomarker assessment of disease activity; PBMC, peripheral blood mononuclear cells; RF, rheumatoid factor; SAA, serum amyloid A; SJIA, systemic juvenile idiopathic arthritis; SRM, selected reaction monitoring; sST2, soluble ST2/IL-1 receptor-like 1; STRING, search tool for the retrieval of interacting genes/proteins platform; TNF, tumour necrosis factor; TTr, transthyretin

## Additional files

Additional file 1: Full summary of included studies. (DOCX 153 kb) Additional file 2: Functional enrichments identified by STRING analysis for the top scoring 15 proteins which differentiated SJIA from non-arthritis conditions or healthy controls. (DOCX 15 kb)
