# Supplementary material for: Review of biomarkers in systemic juvenile idiopathic arthritis: helpful tools or just playing tricks?
Source: Arthritis Res Ther. 2016 Jul 13;18:163. doi: 10.1186/s13075-016-1069-z (PMC4944486; doi:10.1186/s13075-016-1069-z)
Supplement: Additional file 1: — Full summary of included studies. (DOCX 153 kb) [file 13075_2016_1069_MOESM1_ESM.docx]

**Supplemental Table 1: Full summary of included studies**

| **Author and year of study** | **Study aims** | **SJIA + other included patients/ controls/ comparison groups** | **Biomarkers included** | **Method of biomarker measurement** | **Summary of results** | **Diagnostic or Prognostic biomarker described** |
| --- | --- | --- | --- | --- | --- | --- |
| Aggarwal 2007 | To investigate chemokine and their receptors in ERA. | 9 SJIA, 12 PA, 18 HC | CCL5/RANTES and IP-10/CXCL10 | Commercial ELISA | Lower serum IP10/CXCL10 levels were found in SJIA vs ERA. SF concentrations were less than serum for JIA subtypes. | Diagnostic |
| Bica 2007 | To evaluate the distribution and correlation of NO with disease severity in JIA. | 34 SJIA, 34 OA, 29 PA | NO | In-house spectrophotometric assay | NO was greater when disease activity was greater. | Prognostic |
| Bleesing 2007 | To assess if sIL-2Ralpha (sCD25) and sCD163 can diagnose acute MAS complicating SJIA. | 16 SJIA +/- MAS | sCD163 and sIL-2R alpha (sCD25) | Commercial ELISA | sCD163 and aIL-2R alpha were elevated in SJIA-MAS compared with SJIA without MAS. | Diagnostic |
| Bloom 2005 | To determine whether soluble forms of ICAM-1 and E-selectin correlate with clinical measures or other markers of endothelial activation. | 8 SJIA, 10 PA, 10 OA, 30 HC | sICAM-1 and sE-selectin | Commercial ELISA | sICAM-1 was elevated in all JIA subtypes compared with HCs, and was elevated in active vs non-active SJIA. | Prognostic |
| Bloom 2007 | To determine the prevalence of AECA in JIA vs HC | 8 SJIA, 10 PA, 10 OA | AECA | In-house ELISA | AECA was elevated in SJIA compared with HC, and elevated in SJIA compared with OA JIA. | Diagnostic |
| Bloom 2009 | To test if the persistence of D-dimer elevation over long follow-up would signal poor outcome. | 31 SJIA | Fibrin D Dimer | Quantitative commercial assay using latex particles | When SJIA patients with persistent elevation of D-dimer were more likely to have a severe outcome. | Prognostic |
| Bobek 2014 | To determine the presence of HMGB1 and sRAGE in different subtypes of JIA and compared to SLE and HC. | 27 SJIA, 34 OA, 35 PA, 19 SLE, 28 HC | HMGB1, sRAGE | Commercial ELISA | Serum HMGB1 was significantly increased in SJIA vs HC and serum sRAGE was significantly reduced in SJIA vs HC. | Diagnostic |
| Bodman-Smith 2004 | Aimed to determine presence of BiP antibodies according to JIA subtype. | 37 SJIA, 41 OA, 33 persistent OA, 43 PA, 16 HC | Anti-BiP antibody | In-house ELISA | No difference in anti-BIP levels between SJIA and HC , but was elevated in RF-positive PA JIA. | Diagnostic |
| Cangemi 2013 | To clinically validate a commercial kit for the measurement of serum hepcidin in healthy children and pathological conditions including children with SJIA related anaemia. | 19 SJIA, 86 HC, 49 patients (including thalassaemia and 16 iron replacement therapy). | Hepcidin | Commercial ELISA | Serum hepcidin was significantly higher in children with anaemia with SJIA compared to the controls. Hepicidin measurement may discriminate anaemia of inflammatory cause in SJIA from iron deficiency anaemia. | Diagnostic |
| Chen 2002 | To measure sE-selectin and sICAM-1 levels among JIA subtypes and controls, and correlate with disease activity | 12 SJIA, 13 PA, 15 OA, 16 HC | sICAM and sE-Selectin | Commercial ELISA | Both sICAM-1 and sE-selectin were elevated in JSIA compared with HC. sE-selectin was elevated in SJIA compared to OA. | Both |
| Chen 2013 | To further investigate IL-18 in the pathogenesis of SJIA. | 45 active SJIA, 23 inactive SJIA, 20 HC | IL-18 and IL-18BP and IL-18:IL-18BP ratio | Commercial ELISA | Patients with active SJIA had higher IL-18 plasma levels and higher IL-18:IL-18BP ratio compared to inactive SJIA patients and HC. | Both |
| De Benedetti 2000 | To measure circulating levels of sE-selectin, sP-selectin and sICAM-1 in patients with JIA and correlate results with disease activity and cytokine levels. | 42 SJIA, 15 HC, 42 total JIA | sE-selectin and sICAM-1 | Commercial immunno-assays. | Still need full paper Both sICAM-1 and sE-selectin were elevated in active SJIA compared with HC. | Diagnostic |
| de Jager 2007 | To determine plasma cytokine levels using a 30-panel multiplex assay in different subtypes of JIA and controls and correlate with disease activity. | 20 SJIA, 30 OA, 15 PA, 9 Diabetes, 20 HC | Panel of 30 significant diagnostic cytokines discussed in detail in this review are: IL-6, IL-12, IL-18, CCL3, CXCL9, XCXL10, OPG and MIF. | Particle based mulitplex immunoassay (LUMINEX) | IL-6, CCL3, CXCL9, CXCL10, OPG and MIF were significantly raised in plasma of SJIA patients vs controls.  CCL3, IL-18 and IL-12 were elevated in SJIA plasma vs other JIA subtypes. | Diagnostic |
| El-Gamal 2004 | sTM is elevated in RA and therefore hypothesized to be raised in SJIA. | 6 SJIA, 17 PA, 30 HC | sTM | Commercial ELISA | sTM was elevated in SJIA patients compared to other JIA subtypes. | Both |
| El-Sayed 2001 | Evaluated serum and SF aggrecan as a metabolic marker and predictor of cartilage destruction in JIA. | 6 SJIA, 20 PA, 5 OSA, 10 arthritis related to CVD | ACAN | Commercial enzyme based immunoassay | Aggrecan (ACAN) was elevated in SJIA patients compared with HC and elevated in SJIA compared with other CVD. | Diagnostic |
| Ezzat 2011 | Increased serum Galectin-3 is noted in RA and implicated as a inflammatory response regulator and was tested in SJIA | 10 SJIA, 20 PA, 12 extended OA, 8 persistent OA | LGALS3 | Commercial ELISA | Elevated galectin-3 distinguished SJIA from HC but not from PA. | Diagnostic |
| Ferreira 2007 | Aimed to determine levels of IgM and IgA RF by ELISA in JIA and correlated with clinical and laboratory markers. | 38 SJIA, 28 OA, 25 PA, 45 HC | IgA RF and IgM RF | In-house ELISA | IgM+/IgA+ RF is lower in SJIA patients compared with PA. IgM-/IgA+ is elevated in SJIA patients compared to PA. | Diagnostic |
| Foell 2004 | To determine neutrophil activation in JIA by analysing serum S100A12 | 33 SJIA, 53 OA, 38 PA, 74 HCs, 16 bacterial infection | S100A12 | In-house ELISA | S100A12 correlated with disease activity and was significantly elevated in SJIA compared to JIA. | Both |
| Foell 2010 | To test whether patients at risk of flare after methotrexate withdrawal can be identified by S100A8/A9 analysis. | 35 SJIA, 96 persistent OA, 56 extended OA, 152 PA, 10 ERA, 15 PsA | S100A8/A9 | In-house ELISA | S100A8/A9 was prognostic for risk of flaring, relapse rate and time to relapse. | Both |
| Frosch 2009 | To test MRP8/14 as a diagnostic tool for the differentiation of SJIA and systemic infections. | 60 SJIA, 50 HC | S100A8/A9 | In-house ELISA | S100A8/A9 distinguished SJIA from infection. | Diagnostic |
| Galeotti 2008 | To measure survivin as a marker of JIA in plasma | 11 SJIA, 12 OA, 23 PA, 46 HC | SAA, Survivin, COMP and IL-6 | Commercial ELISA | SAA, survivin and IL-6 are elevated in SJIA versus HC. | Diagnostic |
| Gheita 2012 | To assess the level of BAFF and APRIL in different JIA subtypes in relation to disease activity | 20 SJIA, 34 HC, 31 OA, 23 OA | BAFF and APRIL | Commercial ELISA | APRIL and BAFF were higher (but not statistically) in SJIA versus JIA subtypes. BAFF and APRIL correlated with increase in CHAQ and JADAS27 in all JIA patients. | Both |
| Gheita 2013 | Resistin levels were tested in patients with JIA. | 19 SJIA, 33 HC, 28 OA, 21 PA | Resistin | Commercial ELISA | Resistin was elevated in SJIA compared with HC and other JIA subtypes. | Diagnostic |
| Gilliam 2008 | A panel of biomarkers in JIA including COMP were tested for association with disease severity. | 9 SJIA, 17 OA, 18 HC | COMP and ACPA | Commercial ELISA | ACPA is elevated in SJIA compared with HC. COMP and ACPA correlated with disease severity. | Both |
| Gorelik 2013 | To correlate FSTL-1 levels with gene expression, known biomarkers and measures of disease activity in SJIA +/- MAS. | 28 SJIA, 30 HC | FSTL-1 | Commercial ELISA | FSTL-1 was elevated in SJIA patients before treatment. | Prognostic |
| Holzinger 2012 | To test the ability of S100A8/A9 serum concentrations to monitor disease activity in patients with SJIA and stratify patients at risk of relapse. | 52 Active and inactive SJIA | S100A8/A9 | In-house ELISA | Predicted relapse in SJIA and was an indicator of disease activity. | Prognostic |
| Huegle 2014 | To determine whether ANA and rheumatoid factor will develop in patients with SJIA over disease course. | 32 SJIA | ANA | Fluorescence assay | Positive ANA titers developed in patients over time: 8/32 patients were positive at diagnosis, 22/32 at follow up. | Prognostic |
| Ishikawa 2013 | To assess the role of IL-33 and ST2 in the pathogenesis of SJIA and correlation with disease activity and severity. | 24 SJIA, 20 HC, 5 PA, 4 SJIA-MAS | sST2 | Commercial ELISA | sST2 was elevated with increased disease activity in SJIA, and increased in SJIA patients compared with HC. Serum IL-33 concentrations did not statistically significantly differ between HC and SJIA. | Both |
| Jelusic 2007 | To describe serum IL-18 levels in active and inactive JIA. | 17 SJIA, 31 OA, 33 PA, 18 HC | IL-18 | Commercial ELISA | Elevated in SJIA compared with other JIA subtypes. Elevated in active SJIA compared with inactive SJIA. | Diagnostic |
| Kounami 2005 | To assess the clinical features in 9 MAS events, 5 in SJIA. | 5 SJIA-MAS | B2M and soluble IL-2 receptor (sCD25) | Not indicated. | B2M was elevated in SJIA-MAS patients compared to SJIA without MAS. | Prognostic |
| Karagiozoglou-Lampoudi 2011 | Serum ghrelin in JIA and association with anti-TNF therapy, disease activity and nutritional status was evaluated. | 8 SJIA, 19 PA, 23 OA, 2 ERA, 50 HC | Ghrelin | Commercial ELISA | Serum Ghrelin in SJIA was significantly lower compared to HC. | Diagnostic |
| Lehmberg 2013 | To identify measures distinguishing MAS in SJIA from FHL and VA-HLH and to define cut off levels. To evaluate suggested dynamic measures differentiating MAS in SJIA from SJIA flares. | 27 SJIA, 90 FHL, 42 VA-HLH, each patient with SJIA had MAS | Ferritin and sCD25 | Commercial ELISA | Serum sCD25, ferritin and CRP were significantly lower in MAS-SJIA patients compared to FHL or VA-HLH. | Prognostic |
|  |  |  |  |  |  |  |
| Ling 2010 | To identify a protein signature that differentiates patients with SJIA flare from those with quiescent disease. | 17 SJIA, 10 SJIA patients in flare and quiescence, 5 PA | TTR, CFH, APO A1, A2M, GSN, C4, AGP1, ACT, APO VI, SAP, HP, CRP, S100A8/A9, S100A12, SAA | Commercial ELISA | 26 plasma proteins were associated with SJIA flare: 15 were highly significant. Nine biomarkers were further evaluated in ELISA and shown to significantly differentiate flare from quiescent disease in SJIA. | Prognostic |
| Lotito 2007 | To verify the importance of interleukin 18 (IL-18) in the pathogenesis of JIA. | 13 SJIA, 13 PA, 24 OA, 25 HCs | IL-6 and IL-18 | Commercial ELISA | IL-18 was increased in active SJIA vs inactive disease. | Prognostic |
| Maeno 2002 | To further the understanding of IL-18 in the pathogenesis of SJIA. | 29 active SJIA, 29 PA, 18 OA, 10 KD, 33 HC | IL-18 and IL-18BP | Commercial ELISA | Serum IL-18 was significantly higher in SJIA vs other JIA and HC. | Diagnostic |
| Masi 2009 | To assess if OPN is a marker of response to methotrexate at disease onset. | 5 SJIA, 30 PA, 23 OA, 2 PsA | OPN | Commercial ELISA | OPN was elevated in SJIA patients compared with HC. | Diagnostic |
| Muzaffer 2002 | To find a biological basis for clinically distinct JIA subtypes, levels of soluble TNF receptors were measured as an indirect measure of cytokine activity. | 11 SJIA, 13 PA, 10 OA | sTNFR55 and sTNFR75 | Commercial ELISA | Levels of aTNFR55 and sTNFR75 differ by JIA subtype and therefore maybe prognostic for subtypes. IL-1RA was igher in SJIA vs pauciarticular JIA, but not statistically. | Diagnostic |
| Nakajima 2009 | To determine COMP levels in SJIA patients during active and inactive disease. | 11 SJIA, 201 HC | COMP | Commercial ELISA | Decreased levels of COMP were in active compared to remission in SJIA, and was decreased in active SJIA compared with HCs. | Prognostic |
| Ozawa 2012 | To detect the differences between SJIA and PA by measuring serum proteins and radiological findings. | 20 SJIA, 16 PA | Anti-CCP antibody and MMP-3 | Not indicated | MMP-3 was higher in SJIA vs PA. Anti-CCP antibody was lower in SJIA vs PA. | Diagnostic |
| Put 2015 | To study the role of IFNg and IFNg associated cytokines in SJIA, SJIA associated MAS and haemophagocytic lymphohistiocytosis (HLH). | 20 SJIA (10 active, 10 inactive), 2 HLH, 3 SJIA-MAS, 16 HC | IP-10, IL-18BP, indoleamine 2,3-dioxygenase, IL-18, IFNg, IL-6 | Commercial ELISA | IFNg was approximately five times higher in active SJIA vs inactive sJIA or HC. IFNg was higher in HLH vs SJIA. Plasma IL-6 was higher in SJIA (active or inactive) vs HC. Plasma IL-18 was higher in active SJIA vs inactive SJIA, and higher in SJIA vs HC. Plasma IP-10 was higher in HLH patients vs patients with inactive SJIA or HC. IL-18BP was elevated more than 5-fold in HLH vs active SJIA, and was higher in SJIA vs HC. | Diagnostic |
| Reddy 2014 | To investigate the prevalence of clinical and traditional laboratory markers of MAS as well as soluble CD163 and soluble interleukin (IL)-2Ra (CD25) in active SJIA patients. | 33 SJIA, 2 SJIA + MAS, 11 PA | sCD25 and sCD163 | Commercial ELISA | 2 patients with MAS had elevated sCD25 vs PA. However, sCD25 was also raised in almost half patients with SJIA without MAS. | Prognostic |
| Sarma 2008 | To determine RANKL, TIMP and OPG levels in SJIA versus controls. | 13 SJIA, 24 ERA, 22 PA, 8 OA, 3 others. | TIMP, RANKL and OPG | Commercial ELISA | RANKL, TIMP and OPG were elevated in SJIA patients compared to HC. | Diagnostic |
| Shahin 2002 | Circulating IL-6, sIL-2R (sCD25), TNF-alpha and IL-10 was measured in SJIA and PA and correlated with Doppler sonography. | 10 SJIA, 9 PA | IL-6, TNF-alpha, IL-10 | Commercial ELISA | TNF-alpha serum levels were higher in SJIA vs PA. IL-10 and IL-6 were higher in patients with PA vs SJIA. | Diagnostic |
| Shenoi 2015 | Shenoi et al. investigated diagnostic markers for SJIA in children with fever who had SJIA compared with children who did not. | 10 active SJIA, 10 febrile non-SJIA controls | S100A12, S100A8, S100A9, S100A8/A9 | Commercial ELISA | S100 proteins were significantly elevated in SJIA vs control, while PD-1 expression was significantly lower. Procalcitonin, CRP and ESR) were not specific for SJIA. | Diagnostic |
| Shimizu 2010 | To compare the cytokine profiles and kinetics in patients with MAS due to SJIA in both active and inactive disease SJIA without MAS, compared to EBV-induced HLH and KD, and to investigate the significance of IL-18 in the pathogenesis of SJIA. | 5 SJIA-MAS, 10 EBV-HLH, 22 KD, 28 HCs | IL-18 | Commercial ELISA | Serum IL-18 was significantly higher in SJIA-MAS patients than in other HLH groups or HC. | Prognostic |
| Shimizu 2012 | To investigate the role of the CD163/HO-1 axis in SJIA, and pro-inflammatory cytokines (IL-10, IL-18, IL-6, neopterin, soluble TNF-α receptor types I and II) in patients with SJIA complicated by MAS. | 4 patients with SJIA-MAS, 10 HC, 10 KD | Neopterin, HO-1, sCD163 and IL-10 | Commercial ELISA | HO-1, sCD163 and IL-10 were elevated in SJIA-MAS, active and inactive SJIA without MAS in comparison to levels of other pro-inflammatory cytokines, and significantly higher than KD and EBV-HLH. | Prognostic |
| Shin 2008 | Screened children with different subtypes of JIA at presentation for ANA. ANA is prognostic for uveitis in patients with OA, but its prognostic use in other subtypes is not known. | 17 SJIA, 16 OA, 10 PA | ANA | Commercial ELISA | Serum ANA patterns and levels change during follow-up in SJIA and other JIA subtypes. | Prognostic |
| Simonini 2001 | Two endproducts of lipid peroxidation and the formation of antibodies against oxidized low density lipoproteins (Ab oxLDL) in different JIA subsets was investigated. | 14 SJIA, 28 OA, 15 PA | AB-oxLDL | Commercial ELISA | AB-oxLDL are higher in SJIA compared with HC, but similarly elevated in all subtypes of JIA. | Diagnostic |
| Simonini 2005 | Neprilysin, a cell surface enzyme particularly found on neurons, is thought to play a role in inflammation via its action of degrading neuropeptides involved in neurogenic inflammation which terminates their inflammatory effects. | 8 SJIA, 52 HC, 34 OA, 16 PA | CD10 | Commercially available coumarin used in a fluorimetric assay. | Patients with SJIA had lower plasma Neprilysin (CD10) levels compared to HC. | Diagnostic |
| Singh 2012 | The authors previously demonstrated reduced sCD21 in various autoimmune disorders, and here investigated sCD21 and sCD23 in subtypes of JIA. | 20 SJIA, 20 OA, 20 PA | sCD21 and sCD23 | Commercial ELISA | sCD21 was significantly decreased in all JIA subtypes. sCD23 was significantly decreased in PA and SJIA but not OA. | Diagnostic |
| Takahashi 2009 | To determine the serum levels of HO-1 in patients with SJIA compared to HC and other rheumatic diseases- | 56 SJIA, 15 PA, 13 SLE, 25 MTCD, 17 KD, 6 Takayasu aortitis, SJIA-MAS, HC | HO-1 | Commercial ELISA | HO-1 was elevated in SJIA compared with HC and the other disease conditions tested. | Diagnostic |
| Tomoum 2009 | To investigate the value of anti-RA33 for diagnosis of JIA, and its relation to disease activity markers and bone resorption. | 7 SJIA, 44 HC, 18 PA, 9 OA | RA33 | Commercial ELISA | RA33 was elevated in in all SJIA subtypes vs HC, but not elevated in SJIA vs HC. | Diagnostic |
| Urakami 2006 | To investigate COMP as a marker of arthritis and/or growth impairment | 8 SJIA, 6 OA, 10 PA, 82 HCs | COMP | Commercial ELISA | Decreased with greater disease activity in SJIA. | Prognostic |
| Wilson 2010 | To examine the source of FSTL-1, factors inducing its expression in arthritis and whether it’s over-expressed in JIA. | 15 SJIA, 54 OA, 26 PA, 15 HC | FSTL-1 | Commercial ELISA | FSTL-1 was elevated in SJIA patients vs HC, and elevated with increased disease activity in SJIA. | Both |
| Wittkowski 2008 | To investigate whether serum concentrations of S100A12 help in deciding whether to treat patients with FUO with antibiotics or immunosuppressive agents | 60 SJIA, 45 HC, 17 FMF, 18 NOMID, 17 MWS, 40 ALL, 5 AML, 83 systemic infection | S100A12 | In-house ELISA | S100A12 is highly overexpressed in SJIA compared to the other disease groups tested (except FMF). | Prognostic |
| Yilmaz 2001 | To investigate serum levels of cytokines in SJIA during inactive and active disease, and compared to HC and other JIA subtypes. | 8 SJIA, 21 HC, 15 PA, 11 OA. | IL-1b, IL-6, IL-12, IL-8, TNF-alpha | Commercial ELISA | IL-1b was significantly higher in SJIA vs PA, and in SJIA vs OA during inactive and active SJIA disease. IL-1beta was significantly higher in inactive SJIA vs HC. IL-12 was significantly higher in SJIA vs OA during inactive disease. IL-6 was significantly higher in SJIA vs OA and SJIA vs PA during active disease. | Diagnostic |
| *Abbreviations: disease / control groups:* HC: healthy controls, OA: oligoarthritis, PA: polyarthritis, SJIA: systemic-onset juvenile ideopathic arthritis, JIA: juvenile idiopathic arthritis, OSA: osteoarthritis, SLE: systemic lupus erythematosus, ERA: enthesitis-related arthritis, PsA: psoriatic arthritis, RA: Rheumatoid Arthritis, KD: Kawasaki Disease, MAS: macrophage activation syndrome, SJIA-MAS: MAS in patients with SJIA, FHL: familial hemophagocytic lymphohistiocytosis, VA-HLH: virus-associated haemophagocytic lymphohistiocytosis, EBV-HLH: Epstein-Barr virus haemophagocytic lymphohistiocytosis, MTCD: mixed connective tissue disease CVD: collagen vascular disorders (SLE/scleroderma), ELISA: enzyme-linked immunoassay, vs: versus.  *Abbreviations: biomarkers:* A2M: alpha-2-macroglobulin, AB-oxLDL: antibodies to low-density lipoprotein, ACAN: aggrecan core protein, ACPA: anti-citrullinated protein antibodies, ACT: alpha-1-antichymotrypsin, AECA: anti-endothelial cell antibodies, AGP1: alpha-1-acid-glycoprotein, ANA: antinuclear antibody, anti-BIP: anti-immunoglobulin binding protein, anti-CCP: anti-cyclic citrullinated peptide, APO A1: apolipoprotein A1, APO VI: apolipoprotein A VI, APRIL: a proliferation inducing ligand, B2M: beta-2-microglobulin, BAFF: B-cell activating factor, C4: complement C4, CCL3: chemokine (C-C motif) ligand 3, CD10: cluster of differentiation antigen 10, CFH: complement factor H, COMP: cartilage oligomeric matrix protein, CXCL9: chemokine (C-X-C motif) ligand 9, FSTL-1: follistatin-like protein 1, GHRL: ghrelin, appetite regulating hormone, GSN: gelsolin, HMGB1: high mobility group box protein 1, HO-1: heme-oxygenase-1, HP: haptoglobin, IFNG: interferon gamma, IgA RF: immunoglobulin A rheumatoid factor, IgM RF: immunoglobulin M rheumatoid factor, IL-10: interleukin-10, IL-12: interleukin-12, IL-18: interleukin 18, IL-18BP: IL-18 binding protein, IL-1b: interleukin 1b, IL-6: interleukin 6, IP-10/CXCL10: ifng-induced protein 10, or c-x-c motif chemokine 10, LGALS3: galectin-3, MIF: macrophage migration inhibitory factor, MMP-3: matrix metalloproteinase-3, NO: nitric oxide, OPG: osteoprotogerin, OPN: osteopontin, RA33: anti-heterogeneous nuclear ribonucleoprotein A2 antibodies, RANKL: tumour necrosis factor (TNF) ligand superfamily member 11, S100A12: s100 calcium-binding protein A12, S100A8/A9 or MRP8/14: myeloid regulatory protein 8/14 complex, SAA: serum amyloid A, SAP: serum amyloid P, sCD163: soluble cluster of differentiation 163, sCD21: soluble cluster of differentiation 21, sCD23: soluble cluster of differentiation 23, sCD25: soluble cluster of differentiation 25, sE-selectin: soluble E-selectin adhesion molecule, sICAM-1: soluble intracellular adhesion molecule-1, sRAGE: soluble recetor for advanced glycation end products, sST2: soluble ST2, sTM: soluble thrombomodulin, sTNFR55: soluble tumour necrosis factor receptor 55, sTNFR75: soluble tumour necrosis factor receptor 75, TIMP: tissue inhbitors of metalloproteinases, TNF-alpha, tumour necrosis factor-alpha, TTR: transthyretin. | | | | | | |
