# Supplementary material for: Review of biomarkers in systemic juvenile idiopathic arthritis: helpful tools or just playing tricks?
Source: Arthritis Res Ther. 2016 Jul 13;18:163. doi: 10.1186/s13075-016-1069-z (PMC4944486; doi:10.1186/s13075-016-1069-z)
Supplement: Additional file 2: — Functional enrichments identified by STRING analysis for the top scoring 15 proteins which differentiated SJIA from non-arthritis conditions or healthy controls. (DOCX 15 kb) [file 13075_2016_1069_MOESM2_ESM.docx]

| **Biological Processes** n=172 GO terms (gene count > 10 shown only) | | |
| --- | --- | --- |
| pathway ID | pathway description | count in gene set |
| GO:0050794 | regulation of cellular process | 14 |
| GO:0051716 | cellular response to stimulus | 12 |
| GO:0070887 | cellular response to chemical stimulus | 11 |
| GO:0048583 | regulation of response to stimulus | 11 |
| GO:0048522 | positive regulation of cellular process | 11 |
| GO:0044707 | single-multicellular organism process | 11 |
| **Molecular Function** n=8 GO terms | | |
| pathway ID | pathway description | count in gene set |
| GO:0005102 | receptor binding | 10 |
| GO:0050786 | RAGE receptor binding | 3 |
| GO:0005515 | protein binding | 13 |
| GO:0035662 | Toll-like receptor 4 binding | 2 |
| GO:0005125 | cytokine activity | 4 |
| GO:0008201 | heparin binding | 4 |
| GO:0050544 | arachidonic acid binding | 2 |
| GO:0005509 | calcium ion binding | 5 |
| **Cellular component** n=5 GO terms | | |
| pathway ID | pathway description | count in gene set |
| GO:0005615 | extracellular space | 13 |
| GO:0005576 | extracellular region | 13 |
| GO:0044421 | extracellular region part | 12 |
| GO:0009897 | external side of plasma membrane | 4 |
| GO:0070062 | extracellular exosome | 8 |
| **KEGG Pathways**, n=11 | | |
| pathway ID | pathway description | count in gene set |
| 05144 | Malaria | 5 |
| 05143 | African trypanosomiasis | 4 |
| 04060 | Cytokine-cytokine receptor interaction | 5 |
| 04668 | TNF signaling pathway | 4 |
| 05164 | Influenza A | 4 |
| 04623 | Cytosolic DNA-sensing pathway | 3 |
| 05321 | Inflammatory bowel disease (IBD) | 3 |
| 05323 | Rheumatoid arthritis | 3 |
| 05134 | Legionellosis | 2 |
| 04621 | NOD-like receptor signaling pathway | 2 |
| 05132 | Salmonella infection | 2 |

**Table S2**

Functional enrichments in the top scoring 15 proteins identified as differentiating SJIA from non-arthritis conditions or healthy controls. The false discovery rate was < 0.05 for all pathways listed. Adapted from [www.string-db.org](http://www.string-db.org) analysis.
